# Frontal EEG Asymmetry and Attachment Style During Sequential Decision-Making in the Secretary Problem

**DOI:** 10.3390/bs16020275

**Published:** 2026-02-14

**Authors:** Ilan Laufer

**Affiliations:** Department of Industrial Engineering and Management, Ariel University, Ariel 40700, Israel; ilanl@ariel.ac.il

**Keywords:** attachment style, decision-making, EEG, frontal alpha asymmetry, regulatory effort, secretary problem, sequential choice, uncertainty

## Abstract

Sequential decisions often unfold under uncertainty, requiring people to evaluate options one at a time and commit without the possibility of returning to earlier choices. Although such situations appear neutral on the surface, they engage emotional and regulatory processes that vary across individuals. This study examined whether frontal EEG asymmetry during the classic secretary problem is associated with attachment style. Twenty-seven participants completed a sequential decision-making task while EEG was recorded, and analyses focused on asymmetry at frontal sites. Asymmetry was extracted at three points in each decision sequence (start, middle, final), and additional regressions assessed whether deliberation length was related to asymmetry at the moment of choice. Insecure and secure participants showed different patterns of asymmetry across phases, and longer deliberation was linked to greater left-frontal activation. These associations suggest that individual differences related to attachment may be reflected in neural engagement even in abstract, non-emotional tasks. The findings point to frontal asymmetry as a potential dynamic marker of internal regulation during sequential choices and should be interpreted as exploratory.

## 1. Introduction

Many of life’s decisions do not happen all at once. Instead, they unfold gradually. Options appear one at a time, and once passed over, they are gone for good. The secretary problem reflects this familiar dilemma. In it, participants review a series of offers and must choose one, knowing that earlier options cannot be revisited. While the task is abstract, it mirrors real-life situations like hiring, dating, or shopping, where choices must be made under pressure and with limited information (Ferguson, 1989; Skarupski, 2020).

The original version of the task comes from mathematics and optimal stopping theory, where the goal is to identify the best moment to commit (Bearden et al., 2005; Brera & Fu, 2023; Liu et al., 2023; Tickle et al., 2023). But people do not operate like formulas. Emotions, motivation, and personality traits all shape how we navigate uncertainty and decide when to act (Lavín et al., 2024; B. Zhang & Shou, 2022; Epishin & Bogacheva, 2020; Bergquist & Ekelund, 2025). One way to observe these internal processes is through frontal EEG asymmetry, which reflects the balance of alpha activity between the left and right frontal cortex. Since alpha power is inversely related to neural activity, lower alpha suggests greater engagement (Goodman et al., 2013; Müller et al., 2018; Smith et al., 2018; Zhao et al., 2018). From a neurophysiological perspective, the secretary problem offers a controlled framework for examining how frontal regulatory systems are dynamically engaged as uncertainty increases and commitment becomes imminent.

This type of asymmetry has been linked to both emotional responses and cognitive styles. Greater left-frontal activation is often associated with proactive control, sustained attention, and task-focused engagement (Gómez-Ariza et al., 2017; Stewart et al., 2011). In contrast, right-frontal activation tends to reflect reactive control, vigilance, and sensitivity to negative feedback (Xue et al., 2013; Grassini et al., 2020). For instance, individuals with stronger left-frontal activity typically perform better on tasks involving working memory (Pavlov & Kotchoubey, 2022; Spironelli & Borella, 2021), while right-frontal dominance is more common under stress or during error monitoring (Grassini et al., 2020; Moran et al., 2017). These frontal asymmetry patterns also relate to executive functions like inhibition and goal regulation. Although they shift moment to moment depending on the context, they also reflect stable traits (Ambrosini & Vallesi, 2017; Enriquez-Geppert et al., 2014; Grimshaw & Carmel, 2014; Ellis et al., 2017). EEG thus allows us to examine how emotion and regulation unfold as decisions are made.

Accumulating research links these neural dynamics to attachment-related traits. Insecure attachment has been associated with less effective regulation across physiological, neural, and behavioral systems. Elevated stress responses, such as increased cortisol, heart rate, or skin conductance, are often found in insecurely attached individuals (Gander & Buchheim, 2015; Buchheim et al., 2017; Gander et al., 2022; Petrowski et al., 2017) alongside asymmetrical EEG profiles consistent with emotional strain and reduced flexibility in regulation (Rognoni et al., 2008; Kungl et al., 2016; Vrtička & Vuilleumier, 2012).

Recent systematic reviews reinforce this connection, showing that secure attachment is consistently linked to balanced emotion regulation, while insecure dismissing and unresolved attachment styles are associated with physiological stress responses and regulatory breakdowns. Preoccupied individuals tend to amplify emotional expression under stress, while unresolved individuals often fail to contain or integrate emotional distress at all (Eilert & Buchheim, 2023; Tironi et al., 2021; Lorenzini & Fonagy, 2013).

In earlier work using the secretary problem (Mizrahi et al., 2024), our team investigated how attention and effort evolved during the task, using event-related potentials and the Theta/Beta ratio. That study revealed shifting cognitive load over time but did not examine hemispheric asymmetry. The present study builds on those findings by focusing on frontal EEG asymmetry as a marker of emotional and regulatory differences. Attachment style may play a critical role here. Secure individuals tend to remain balanced under pressure, while those with insecure attachment, whether anxious, avoidant, or unresolved, are more likely to become dysregulated or withdraw in demanding situations (Vrtička & Vuilleumier, 2012; Eilert & Buchheim, 2023; Lorenzini & Fonagy, 2013; Gardner et al., 2020; Clear et al., 2019). These tendencies show up in EEG patterns. Insecure attachment is often associated with greater right-frontal activation under stress or ambiguity (Gander & Buchheim, 2015; Rognoni et al., 2008; Kungl et al., 2016; Becske et al., 2023; Biro et al., 2021). Avoidant individuals typically display right-frontal dominance and downplay positive cues (Rognoni et al., 2008; Kungl et al., 2016; Becske et al., 2023), while anxious individuals may show left-frontal dominance but heightened reactivity (Rognoni et al., 2008; Kungl et al., 2016; Becske et al., 2023). These differences emerge early and persist across development, from infancy to adolescence to adulthood (Gander & Buchheim, 2015; Kungl et al., 2016; Biro et al., 2021; Howarth et al., 2016; Puhlmann et al., 2023; Lyons-Ruth et al., 2016; McConnell & Moss, 2011).

Because attachment style shapes how individuals regulate uncertainty, effort, and commitment under pressure, it is expected to influence how decisions unfold over time in sequential decision-making tasks such as the secretary problem. Yet it remains unclear whether these attachment-linked EEG patterns also appear in tasks without explicit emotional content. This study asks whether frontal asymmetry during the secretary problem, a decision-making task with no overt emotional triggers, varies by attachment style. To test this, we proposed two hypotheses. First (H1), we expect secure and insecure individuals to show different frontal asymmetry trajectories as the task progresses. Specifically, insecure participants are expected to display greater right-frontal activation near the point of commitment as decisions become imminent (Gander & Buchheim, 2015; Kungl et al., 2016). Second (H2), within the insecure group, longer deliberation, operationalized as the number of offers reviewed, is expected to be associated with stronger right-frontal asymmetry at the moment of choice (Ravaja et al., 2016; Nelson et al., 2014). This approach combines structured decision-making with attachment neuroscience to clarify how the brain manages uncertainty and commitment in real time.

## 2. Materials and Methods

### 2.1. Participants

Participant recruitment followed a two-stage process. In the first stage, 96 individuals completed the 36-item ECR-R questionnaire, which was used to operationalize attachment style. From this initial cohort, 27 participants were selected and invited to the EEG laboratory for the second stage, where they performed the secretary problem task while their EEG activity was recorded.

The EEG sample comprised 27 undergraduate engineering students (16 women and 11 men) with a mean age of 23.8 years (men: 24.9 ± 2.6; women: 23.06 ± 1.8). A proportional allocation approach was used to preserve the relative representation of the attachment categories within the EEG sample. This procedure maintained the same distribution observed in the larger pool. Accordingly, the final group included six securely attached participants, nine with anxious attachment, seven with avoidant attachment, and five classified as fearful-avoidant. All participants were right-handed, producing a group that was highly consistent in age, educational level, and handedness. Although age and gender were not included as covariates in the analyses, the restricted variability in these characteristics reduces the likelihood of meaningful confounding effects. The two-stage sampling procedure was used to identify participants with clearly defined attachment profiles prior to EEG recording, thereby increasing sensitivity to attachment-related differences in neural activity. Proportional allocation was applied to preserve the relative distribution of attachment categories observed in the larger screening sample. For the purposes of the present analyses, anxious, avoidant, and fearful-avoidant participants were grouped into a single insecure attachment category. This decision follows established attachment research distinguishing secure versus insecure regulatory patterns and was adopted to support interpretable group-level comparisons. Ethical approval was granted by the institution’s IRB, and all participants provided written informed consent before taking part.

### 2.2. Task Procedure

The secretary problem was implemented as a sequential decision-making task, in which participants were instructed to choose the best offer (e.g., salary) from a list presented one item at a time. Unlike standard decision-making tasks in which options are presented simultaneously or can be revisited, the secretary problem requires irreversible sequential evaluation, such that each rejection permanently eliminates an option and increases commitment pressure over time. Offers were drawn randomly from a known numerical range (1–100), and once rejected, an offer could not be revisited. The total number of offers per list was known in advance. Participants had to decide immediately whether to accept or reject each offer. The reward for each trial was the value of the selected offer.

Each participant completed six blocks of the task, with varying memory demands: two blocks each of maximum list lengths of 10, 15, and 20 offers, presented in randomized order. The ordinal position of the chosen offer was recorded for each trial and used to quantify both decision timing and deliberation trajectory length.

### 2.3. EEG Recording and Preprocessing

EEG activity was collected with a 16-channel active amplifier system (USBAMP, g.tec, Austria) at a sampling rate of 512 Hz, using the standard 10–20 electrode layout. The initial sampling rate of 512 Hz was used to ensure accurate acquisition of EEG signals and to support reliable artifact identification during preprocessing. For subsequent wavelet-based analysis of alpha-band activity, the data were downsampled to 64 Hz, which is sufficient to represent frequencies up to 32 Hz while reducing data dimensionality and improving computational efficiency without loss of information relevant to the present analyses. Impedance levels were kept below 5 kΩ for all electrodes. The analysis concentrated on six frontal and prefrontal sites (Fp1, F7, Fp2, F8, F3, F7). These locations were selected due to their frequent use in studies of emotional and cognitive processes and their relevance for attachment-related research (Robble et al., 2021; Singh & Singh, 2021; J. Zhang & Chen, 2020; Y. Zhang et al., 2020). EEG measures were not used to classify attachment style, but to examine neural correlates of regulatory engagement during the task. Frontal and prefrontal regions are broadly involved in emotion regulation and higher-order control functions, which are central to understanding how attachment patterns shape cognitive performance. The raw EEG data were filtered with a 1–30 Hz bandpass filter and a 50 Hz notch filter to suppress slow drifts, high-frequency noise, and power-line interference.

Artifact correction was performed using Independent Component Analysis (ICA). ICA separates multichannel recordings into statistically independent components under the assumption of non-Gaussian and independent signal sources. In EEG applications, this procedure is routinely used to identify and remove components associated with eye movements, muscle activity, and other non-neural sources of interference (Pester & Ligges, 2018). By removing such components, the method yields a cleaner representation of the underlying neural activity (Hyvärinen & Oja, 2000; Vigário et al., 2002). Independent components were inspected and rejected based on established EEG criteria, including scalp topography, time-course characteristics, and frequency content indicative of ocular or muscle artifacts. Component rejection was performed consistently by a single trained analyst.

Epochs were time-locked to the participant’s decision click (response onset), extending from −1000 ms to +200 ms relative to the response. This window was selected to capture both anticipatory neural activity preceding the decision and immediate post-decision processing. The average latency between the onset of the final offer and the decision click was approximately 1.2 s, ensuring minimal overlap between the final stimulus presentation and the epoch.

Alpha-band activity (8–13 Hz) was extracted using a three-level discrete wavelet transform (DWT) (Shensa, 2002; Hazarika et al., 1997), which preserved both spectral and temporal resolution. The DWT was implemented using the Daubechies-4 (db4) mother wavelet. The DWT decomposes the EEG signal into several scales. Each decomposition level applies a low-pass filter h(n) and a high-pass filter g(n), followed by down-sampling by a factor of 2. In this study a 3-level DWT was used (Mizrahi et al., 2022). The EEG data were sampled at 64 Hz. At this sampling rate, a 3-level dyadic decomposition yields detail bands corresponding approximately to 16–32 Hz (D1), 8–16 Hz (D2), and 4–8 Hz (D3); accordingly, alpha activity (8–13 Hz) was derived from the level-2 detail coefficients (D2). A 3-level decomposition is appropriate for this sampling rate because it separates the lower frequency components while maintaining adequate temporal resolution. After three levels the approximation component corresponds to activity below approximately 4 Hz and the detail components represent progressively higher frequency ranges. This provides a structured frequency breakdown of the EEG signal for subsequent analysis without loss of relevant information. For each epoch, alpha power was calculated as the squared magnitude of the DWT coefficients and averaged over time. Left hemisphere alpha power was defined as the mean power across electrodes F3, F5, and F7; right hemisphere alpha power was defined similarly across F4, F6, and F8. EEG epochs were baseline-corrected at the signal level prior to wavelet decomposition, and frontal alpha asymmetry was computed as a within-participant left–right comparison across homologous electrode pairs.

Frontal EEG asymmetry was computed as the natural logarithm of right alpha power minus the natural logarithm of left alpha power (Rognoni et al., 2008):

Asymmetry = ln(Right Alpha Power) − ln(Left Alpha Power)

Positive values indicate relatively greater left-frontal activation (associated with approach motivation), while negative values reflect greater right-frontal activation (typically linked to withdrawal-related processes).

### 2.4. Data Analysis

#### 2.4.1. Phase-Based Asymmetry Analysis

Frontal asymmetry was extracted at three predefined points in the decision sequence. The first phase corresponded to the first offer presented. The second phase corresponded to a middle point in the viewing sequence and was defined for each trial as the offer located halfway through the set of viewed options. Importantly, this midpoint was defined relative to the actual number of offers viewed in each trial, rather than the maximum possible list length of the block. The third phase corresponded to the final offer chosen.

Decision phases were defined to reflect distinct stages of the secretary problem, corresponding to early exploration, mid-sequence evaluation, and imminent commitment. This segmentation follows the sequential structure of the task, in which decision-relevant demands change as the remaining options decrease. Asymmetry values were averaged across trials within each phase to obtain participant-level estimates aligned with sustained regulatory engagement rather than trial-specific fluctuations.

These three phases (start, middle, final) were treated as a within-subject factor with three levels. Attachment Style (secure or insecure) was included as a between-subject factor. A 3 × 2 mixed-design ANOVA was conducted to test whether asymmetry varied across phases and whether these variations differed between attachment groups. The homogeneity-of-variance assumption for the between-subject factor (Attachment Style) was evaluated using Levene’s test separately for the Start, Middle, and Final decision phases (α = 0.05). No violations were detected in any phase.

#### 2.4.2. Trajectory-Length Analysis

A second analysis examined whether frontal asymmetry at the decision point depended on the number of offers viewed before choosing. Decision length was defined as the total number of offers a participant viewed before making a selection. Decision length reflects the participant’s stopping point within a trial and is independent of task performance or reward value. Participants could terminate the sequence at any position within each block, resulting in substantial variability in decision length within each list-length condition. Linear regressions were run separately for the secure and insecure groups, with decision length as the predictor and asymmetry at the final decision point as the outcome. This analysis tested whether longer deliberation was linked to different frontal activation patterns as a function of attachment style.

## 3. Results

### 3.1. Phase-Based Asymmetry Analysis

A 3 (Decision Phase: Start, Middle, Final) × 2 (Attachment Style: Secure, Insecure) mixed-design ANOVA was conducted to examine how frontal asymmetry changed across the decision sequence. The analysis showed a significant main effect of Attachment Style, F(1, 25) = 30.17, *p* < 0.0001, η^2^_p_ = 0.55, indicating higher asymmetry values in the insecure group. There was also a significant main effect of Decision Phase, F(2, 50) = 91.33, *p* < 0.0001, η^2^_p_ = 0.79, reflecting systematic variation across the three stages. The interaction between Decision Phase and Attachment Style was significant, F(2, 50) = 10.59, *p* < 0.0002, η^2^_p_ = 0.30, showing that the pattern of change differed between attachment groups. The homogeneity of variance assumption for the between-subject factor (Attachment Style) was explicitly tested using Levene’s tests conducted separately for the Start, Middle, and Final decision phases, and no violations were detected.

**Figure 1:**
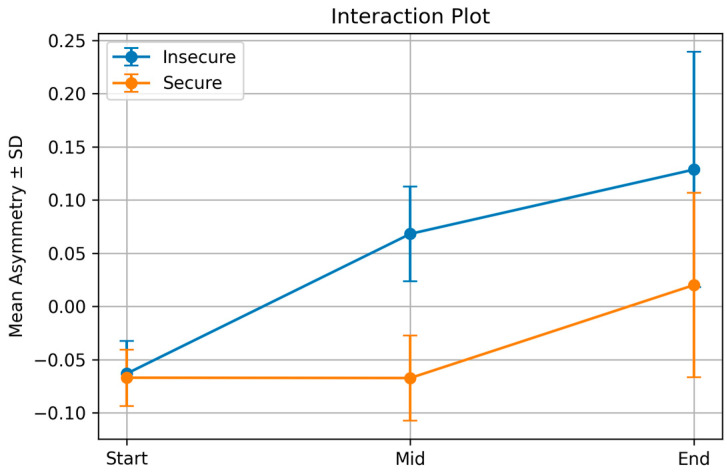
Frontal asymmetry across decision phases by attachment style. Mean asymmetry values are shown for the Start, Middle, and Final stages of the decision sequence for secure and insecure participants. The Middle value corresponds to the asymmetry extracted from the offer located halfway between the first and final viewed options in each trial. Error bars represent standard errors of the mean.

As shown in Figure 1, both groups began the task with similar and slightly negative asymmetry values at the Start phase. At the Middle phase the groups had diverged sharply, with the insecure group showing a large increase and the secure group remaining near the same negative level observed at Start. Between the Middle and Final phases both groups showed an upward change of similar magnitude. However, because the insecure group began the final phase from a higher level, their asymmetry remained substantially higher overall. Thus, the insecure group showed a large increase from Start to Middle and a further rise toward the Final stage, whereas the secure group showed little change until the final phase, where a noticeable increase appeared.

Overall, the magnitude of change across phases was substantially larger in the insecure group. This pattern indicates that asymmetry varied more across phases for insecure participants, whereas secure participants remained relatively close to baseline throughout the task.

### 3.2. Trajectory-Length Analysis

**Figure 2:**
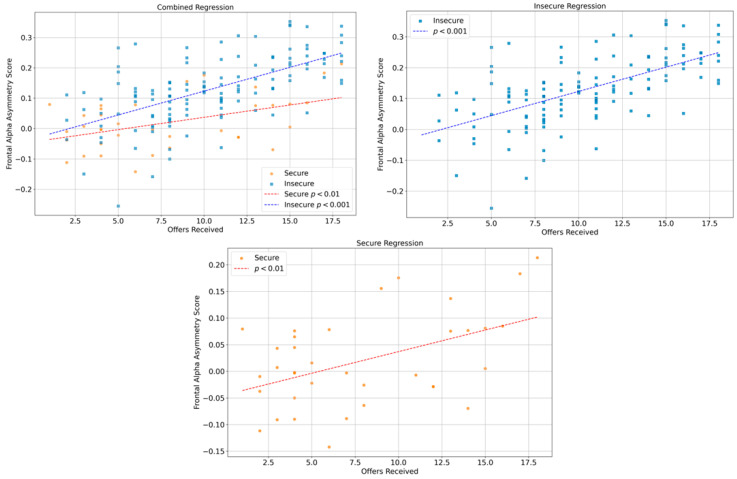
Regression plots illustrating the relationship between deliberation length (offers viewed) and frontal EEG asymmetry at the decision point, shown for the combined sample (**top left**), insecure participants (**top right**), and secure participants (**bottom**).

To assess whether decision length predicted frontal EEG asymmetry at the moment of choice, separate linear regressions were conducted for participants with secure and insecure attachment styles. Figure 2 shows the regression analysis in which asymmetry at the final offer served as the dependent variable and the number of offers reviewed served as the predictor. The figure is arranged in three panels that display the combined sample, the insecure group, and the secure group.

The top-left panel presents the combined regression across all participants. In both attachment groups, the number of offers reviewed is positively associated with final-phase frontal EEG asymmetry, with a steeper slope observed for the insecure group. The top-right panel shows the regression for insecure participants. The data exhibit a clear upward trend, indicating that longer deliberation (i.e., reviewing more offers) is associated with higher asymmetry values at the final decision point (β = 0.01569, *p* < 0.001). The bottom panel displays the secure participants. Although the data are more dispersed, the relationship remains positive and statistically significant, with a smaller effect size relative to the insecure group (β = 0.00812, *p* = 0.00382).

Taken together, the three panels show that longer deliberation predicts increased left frontal activation in both groups, with a much stronger effect among insecure individuals. This suggests that deliberation length modulates neural asymmetry more strongly in the insecure group, consistent with the interpretation that they experience greater cognitive or emotional load as decisions unfold.

The magnitude of the reported effect sizes should be interpreted with caution given the modest sample size. The present estimates describe the pattern observed in the current sample and warrant confirmation in larger samples.

## 4. Discussion

This study examined how attachment style relates to frontal EEG asymmetry during sequential decision-making in the secretary problem task. We tested two hypotheses: first, whether secure and insecure individuals show different asymmetry patterns across decision stages (Kungl et al., 2016; Biro et al., 2021; Chen, 2025). Second, whether longer deliberation predicts stronger asymmetry at the point of choice among insecure individuals (Gander & Buchheim, 2015; Buchheim et al., 2017; Revilla et al., 2024). Both hypotheses were supported, although the direction of the effects differed from what we initially expected.

### 4.1. Hypothesis 1: Asymmetry Across Decision Phases

In line with the first hypothesis, a significant interaction emerged between decision phase and attachment style. Insecure participants showed a rising pattern of asymmetry across the three phases. Their values increased from the Start phase to the Mid phase and increased again at the End phase. Secure participants showed only small changes across the task. They began slightly negative, became somewhat more negative at the Mid phase, and then showed a slight increase at the End phase. Overall, the secure group remained close to baseline.

This pattern reflects a larger phase-related modulation of frontal asymmetry in insecure participants compared to secure participants, consistent with reports linking attachment insecurity to heightened neural engagement under increasing task demands (Kungl et al., 2016; Laufer et al., 2024; Leyh et al., 2016).

This result diverges from earlier predictions that insecurity would be linked to right-frontal activation, often associated with avoidance or emotional withdrawal (Kungl et al., 2016). Instead, insecure participants seemed to become more cognitively engaged as the task progressed (Van Leeuwen et al., 2025). The rise in left-frontal activation could reflect a growing effort to manage internal discomfort or to maintain control under pressure (Gander & Buchheim, 2015). The stable pattern seen in the secure group suggests that they were less affected by the task’s unfolding demands and were better able to regulate consistently (Gander et al., 2022).

This interpretation resonates with past findings that suggest insecure individuals show elevated neural engagement in response to increased internal uncertainty, even in emotionally neutral tasks (X. Zhang et al., 2018). Such patterns may reflect compensatory effort aimed at managing cognitive load, especially when regulatory demands accumulate across phases of a decision sequence (Hiebler-Ragger et al., 2021). Similar dynamics have been reported in insecurely attached individuals performing tasks involving exclusion or feedback, where increased frontal control activation reflects regulatory strain rather than disengagement (Eilert & Buchheim, 2023). Furthermore, the shift toward effortful cognitive engagement is consistent with broader models of attachment-related control, which suggest that insecure individuals often rely on prefrontal regulation to inhibit affect and maintain behavioral organization (Moutsiana et al., 2014).

Notably, a recent resting fMRI study demonstrated that trait-like attachment anxiety is associated with increased amplitude of low-frequency fluctuations and stronger connectivity within emotion-regulation networks, reflecting a baseline predisposition for heightened self-regulatory engagement even in the absence of task demands (Deng et al., 2021). Our task-based findings extend this by showing that such predispositions are mobilized dynamically when decision stress increases.

A related finding from resting EEG work showed that individuals who habitually use cognitive reappraisal, an effortful regulatory strategy, display stronger left-frontal activation even at rest, indicating that regulatory habits can shape trait-level asymmetry profiles (Kelley & Hughes, 2019; Yang et al., 2021; Li et al., 2024). This further supports the idea that insecure individuals may engage in habitual compensatory regulation across both resting and task contexts. Importantly, some studies (Goodman et al., 2013; Berretz et al., 2022) showed that trait-level frontal asymmetry may interact with transient states such as challenge or threat to dynamically influence moment-to-moment regulation. This aligns with our interpretation that insecure individuals engage a trait-based regulatory orientation, which is then selectively amplified during task-related stress or cognitive demand.

### 4.2. Hypothesis 2: Decision Length and Final Asymmetry

According to our second hypothesis, insecure individuals who spent more time reviewing options would show stronger right-frontal activation at the moment of choice. However, the direction of the result was unexpected. Insecure participants who took longer to decide actually showed stronger left-frontal activation at the final decision point, a pattern that was statistically significant. Although a similar association appeared in the secure group, it was much smaller.

Taken together, these findings suggest that longer deliberation may lead insecure individuals to increase their regulatory engagement rather than shut down. The increased left-frontal activation likely reflects a sustained effort to manage uncertainty and maintain control under growing cognitive demand. Rather than withdrawing, these individuals appear to stay engaged as the task becomes more demanding, whereas secure individuals show a weaker link between decision length and asymmetry, suggesting that extended deliberation carries less emotional cost for them. This interpretation aligns with evidence that insecure individuals show increased frontal engagement during emotionally or cognitively challenging tasks, reflecting compensatory regulatory effort rather than avoidance (Goodman et al., 2013). Moreover, longer decision times have been linked to greater frontal asymmetry across uncertain decision contexts, consistent with sustained cognitive mobilization rather than withdrawal (Revilla et al., 2024).

At a neural level, the activation patterns may index an ongoing struggle to resolve ambiguity and maintain performance under uncertainty, even in the absence of overt affective threat. This interpretation aligns with evidence that insecure individuals show sustained frontal activation across difficult cognitive tasks, reflecting heightened cognitive control demands and a mismatch between perceived control and task difficulty (X. Zhang et al., 2018). Moreover, insecure individuals may also experience heightened vigilance and rumination, extending their engagement with decision-relevant information. Supporting this interpretation, resting EEG research has shown that higher attachment anxiety is associated with stronger leftward frontal asymmetry, a marker of preemptive control and sustained regulatory activation (Verbeke et al., 2014). Similarly, the habitual use of cognitive reappraisal, rather than suppression, predicts stronger left-frontal asymmetry at rest, suggesting that trait regulatory styles influence baseline neural organization (Kelley & Hughes, 2019).

Taken together, these converging findings indicate that the sustained asymmetry observed in insecure individuals during high-effort decisions may represent the dynamic activation of enduring regulatory tendencies under stress.

### 4.3. Interpretation of Asymmetry Patterns

Regarding the interpretation of asymmetry patterns, frontal asymmetry was defined as the natural log difference in alpha power between left and right frontal sites, with higher values indicating stronger left-frontal activation. This index has been linked to executive control, motivational engagement, and sustained cognitive effort during decision-making (Revilla et al., 2024). While left-frontal asymmetry has also been associated with approach motivation, its functional meaning depends strongly on task context and affective framing. In the present task, which involved abstract numerical rewards and no explicit emotional or social cues, together with the scaling of asymmetry with deliberation length, left-frontal asymmetry is more plausibly interpreted as reflecting sustained regulatory engagement rather than reward-driven approach.

Furthermore, the absence of right-frontal shifts suggests that this neutral, abstract task may not trigger the avoidance responses typically seen in emotionally charged contexts. Instead, insecure individuals may respond to cognitive pressure with increased effort rather than emotional withdrawal. These patterns mirror prior reports of leftward asymmetry during non-affective cognitive load in insecure populations, supporting the interpretation of compensatory control recruitment rather than disengagement. This interpretation is consistent with evidence that insecure attachment is associated with heightened and sustained activation in anterior regions, even when task demands are ambiguous or abstract, reflecting effortful attempts to regulate and maintain performance (Gander & Buchheim, 2015).

In addition, in resting-state EEG, insecure attachment, particularly anxiety, has been linked to an underlying leftward bias, which may reflect a trait-level readiness to engage in top-down control regardless of external triggers (Verbeke et al., 2014). A similar pattern has been observed among individuals who habitually use cognitive reappraisal, where sustained left-frontal EEG activity at rest reflects chronic regulatory engagement even without overt emotional challenge (Kelley & Hughes, 2019). Moreover, evidence suggests that the influence of baseline asymmetry patterns is context-dependent, with task-related features either amplifying or reducing pre-existing asymmetry depending on motivational framing, highlighting that left-frontal activation represents an adaptive interaction between enduring predispositions and situational regulatory demands (Goodman et al., 2013).

### 4.4. Theoretical Implications

These results add to the growing body of work on attachment and neural regulation by showing that differences in frontal asymmetry are visible even in tasks that lack explicit emotional content. Insecure individuals appear to invest more regulatory effort as they move toward a decision, while secure individuals maintain a steadier baseline. This pattern reflects deeper differences in how people cope with uncertainty and control demands (Eilert & Buchheim, 2023).

Furthermore, the findings suggest that attachment-related regulation strategies are not limited to social or emotional situations. They shape how individuals manage internal stress during abstract tasks, reinforcing the idea that attachment influences not only relationships but also general patterns of self-regulation. This broader view of attachment-based neural regulation aligns with recent neuroimaging evidence showing similar dynamics in non-interpersonal problem-solving and decision-making contexts, highlighting attachment as a stable trait that interacts with cognitive control systems across diverse domains (Eilert & Buchheim, 2023).

In addition, the findings suggest that the chronic engagement of cognitive regulatory strategies, particularly in the absence of external affective cues, is a core component of insecure attachment, especially among those high in attachment anxiety. Resting-state neuroimaging shows that the neural architecture supporting frontal control remains tonically active in these individuals, reflecting a trait-level regulatory stance (Deng et al., 2021). Our task-based results align with recent EEG findings that demonstrate how this same control network becomes dynamically engaged during cognitive regulation demands, linking trait neural patterns with situational effort (Domic-Siede et al., 2025).

Finally, recent evidence suggests that frontal EEG asymmetry indexes more than stable attachment-related traits. It also reflects how individuals habitually regulate emotion and effort across contexts. Resting asymmetry patterns have been linked to preferred regulation strategies, forming a behavioral–neural bridge that connects enduring dispositions with adaptive control mechanisms (Palmiero & Piccardi, 2017). Consistent with this, studies show that individual differences in trait asymmetry influence how effectively people recruit regulatory resources when facing challenge, highlighting asymmetry as a flexible marker that integrates stable biases with moment-to-moment control demands (Goodman et al., 2013).

### 4.5. Limitations

This study has several limitations that should be considered when interpreting the results. First, the modest sample size limits statistical power and constrains the precision of effect size estimates. Although clear patterns emerged, the present findings should be interpreted cautiously and viewed as preliminary, pending replication in larger and more diverse samples. Second, all insecure participants were grouped into a single category, which may have obscured meaningful differences between subtypes such as anxious and avoidant. Each subtype may involve distinct neural and regulatory mechanisms, and future research should examine them separately to clarify their unique patterns. Recent EEG evidence shows that attachment anxiety and avoidance are associated with divergent connectivity and activation profiles during emotion regulation tasks, where anxious individuals tend to show heightened frontal engagement and reduced theta coherence during reappraisal, whereas avoidant individuals exhibit increased beta activity during suppression, consistent with inhibitory control efforts (Domic-Siede et al., 2025). Similarly, ERP research indicates that attachment anxiety, rather than avoidance, is linked to heightened neural responsiveness during regulatory challenges, as reflected in increased late positive potential amplitudes (Ramos-Henderson et al., 2024). Together, these findings highlight the importance of distinguishing between insecure subtypes when examining attachment-related neural regulation.

Third, while frontal asymmetry provides a useful window into neural dynamics, it does not allow clear separation of the underlying psychological processes. Signals reflecting cognitive effort, arousal, and motivation often overlap, and without behavioral or self-report measures to supplement EEG data, it becomes difficult to determine what drives the observed changes. This interpretive limitation has been widely acknowledged in research (Smith et al., 2017; Harmon-Jones & Gable, 2018; Kaack et al., 2020).

Finally, although our analysis focused on the period surrounding the final decision, this approach likely missed finer-grained neural changes that unfold throughout the task. More detailed temporal analyses could clarify how regulatory engagement evolves moment to moment within a trial. Recent EEG studies have shown that meaningful shifts in control and decision states can occur within very short time windows, showing that such dynamics are visible only when neural data are examined at a finer temporal scale (Nakuci et al., 2023; Pedroni et al., 2017; Jollans et al., 2017).

### 4.6. Future Directions

Future work should examine whether different forms of insecurity show distinct neural signatures and how these relate to moment-to-moment experiences of affect, confidence, or perceived difficulty. Including trial-level behavioral or self-report measures would help connect neural asymmetry with subjective regulatory effort (Smith et al., 2017).

Future studies will also require larger samples and designs that allow attachment subtypes to be examined separately, in order to improve statistical power and interpretive precision. In addition, such work could complement parametric analyses with nonparametric permutation or bootstrap procedures to assess the robustness of asymmetry effects under fewer distributional assumptions.

Methodologically, new analytic approaches such as source localization, time–frequency decomposition, or real-time neural classification could provide a more detailed view of how regulatory engagement unfolds over time. Combining EEG with fine-grained behavioral indicators (e.g., gaze or facial expressions) may also clarify how individuals balance automatic and controlled strategies during decision making (Harmon-Jones & Gable, 2018).

In addition, longitudinal and intervention studies are needed to determine whether these asymmetry patterns change with experience or therapeutic practice, and whether baseline asymmetry serves as a stable trait marker of self-regulatory style. Evidence linking habitual emotion regulation strategies to resting EEG asymmetry suggests that such neural indices may help identify who benefits most from specific forms of training or support (Kelley & Hughes, 2019).

Finally, while continuous time–frequency analyses are well suited for characterizing fine-grained neural dynamics, the phase-based strategy employed in this study was chosen to align with the sequential structure of the secretary problem and the study’s focus on decision-relevant stages. Future work could build on the present approach by integrating phase-based analyses with continuous time–frequency methods to examine within-phase neural dynamics and moment-to-moment regulatory fluctuations as decisions unfold.

### 4.7. Conclusions

Frontal EEG asymmetry during decision-making appears to vary systematically with attachment style. Insecure individuals, especially those who deliberate longer, show stronger left-frontal activation, likely reflecting heightened regulatory effort and cognitive engagement, whereas secure individuals maintain more stable neural profiles across the task (Hiebler-Ragger et al., 2021). These findings suggest that attachment influences how people manage uncertainty even in the absence of explicit emotional content and are consistent with EEG research showing that attachment-related traits modulate regulatory engagement across both social and nonsocial contexts.

The results also align with theoretical models proposing that insecure attachment involves more effortful cognitive control to manage internal conflict, often at the cost of flexibility or efficiency (Moutsiana et al., 2014). Furthermore, resting-state findings link attachment anxiety with sustained left-frontal activation, suggesting a trait-like tendency toward active regulation even without external demands (Deng et al., 2021). In parallel, habitual use of cognitive reappraisal has been associated with similar asymmetry profiles, implying shared neural mechanisms between attachment and regulatory habits (Kelley & Hughes, 2019).

Taken together, these patterns indicate that frontal asymmetry reflects both stable self-regulatory traits and dynamic task demands, supporting a view of emotion regulation as a temporally evolving process embedded in context (Smith et al., 2017). These findings should be regarded as preliminary and intended to motivate further investigation in larger and more diverse samples.

## Data Availability

The experimental data, including electrophysiological recordings and corresponding task logs, are stored on secure servers at Ariel University. Data are available upon reasonable request from the Ariel University Ethics Committee or the author.
